# Application of microfluidic chips in anticancer drug screening

**DOI:** 10.17305/bjbms.2021.6484

**Published:** 2021-09-30

**Authors:** Xin-yue Fan, Zhuo-fen Deng, Yan-yan Yan, Valerii E. Orel, Andrii Shypko, Valerii B. Orel, Donika Ivanova, Christian Pilarsky, Jing Tang, Zhe-Sheng Chen, Jian-ye Zhang

**Affiliations:** 1 Key Laboratory of Molecular Target and Clinical Pharmacology and the State and NMPA Key Laboratory of Respiratory Disease, School of Pharmaceutical Sciences and The Fifth Affiliated Hospital, Guangzhou Medical University, Guangzhou, China; 2 Institute of Immunology, School of Medicine, Shanxi Datong University, Datong, China; 3 Medical Physics and Bioengineering Research Laboratory, National Cancer Institute, Kyiv, Ukraine; 4 Faculty of Biomedical Engineering, National Technical University of Ukraine “Kyiv Polytechnic Institute”, Kyiv, Ukraine; 5 Department of Pharmacology, Animal Physiology and Physiological Chemistry, Faculty of Veterinary Medicine, Trakia University, Stara Zagora, Bulgaria; 6 Department of Surgery, Friedrich-Alexander University of Erlangen- Nuremberg and University Hospital of Erlangen, Erlangen, Germany; 7 Institute for Molecular Medicine Finland (FIMM), Faculty of Medicine, University of Helsinki, Helsinki, Finland; 8 Department of Pharmaceutical Sciences, College of Pharmacy and Health Sciences, St. John’s University, Queens, NY, United States

**Keywords:** Microfluidic chip, anticancer drug screening, high throughput screening

## Abstract

With the continuous development of drug screening technology, new screening methodologies and technologies are constantly emerging, driving drug screening into rapid, efficient and high-throughput development. Microfluidics is a rising star in the development of innovative approaches in drug discovery. In this article, we summarize the recent years’ progress of microfluidic chip technology in drug screening, including the developmental history, structural design, and applications in different aspects of microfluidic chips on drug screening. Herein, the existing microfluidic chip screening platforms are summarized from four aspects: chip structure design, sample injection and drive system, cell culture technology on a chip, and efficient remote detection technology. Furthermore, this review discusses the application and developmental prospects of using microfluidic chips in drug screening, particularly in screening natural product anticancer drugs based on chemical properties, pharmacological effects, and drug cytotoxicity.

## INTRODUCTION

Microfluidic chip technology, also termed micro total analysis systems or “labs on a chip,” has developed rapidly in recent years. Microfluidic cell culture can control micro- and nano-liter fluid flow in precisely defined geometry, and facilitate simultaneous operation and analysis of cells cultured on the fully integrated and automated chips from a single cell level to a large cell population. Microfluidic chips integrate the functions of a whole laboratory into a microchip, including sampling, dilution, reagent addition, reaction, separation, and detection. Thus, microfluidic chip reduces the cost of drug analysis, enriches the ability of drug analysis or detection, makes drug analysis miniaturized and integrated [1,2].

Currently, cancer remains the most devastating disease worldwide. Due to the resistance of cancer stem cells to conventional treatments, the majority of cancer patients have to face recurrence and metastasis after receiving conventional anticancer therapy [3]. In practice, it requires three stages for novel drugs to pass from the primary screening to full approval: the preclinical research stage *in vitro*, the preclinical stage of animal experiments *in vivo*, and the clinical trial stage with human participation [4]. Microfluidic drug screening is a new technology with an extensive use in the screening of anticancer drugs [5]. Although animal tests are essential for preclinical screening in the process of drug discovery, there are limitations, such as ethical considerations and species differences. To circumvent these issues, cell-based assays using human cells have been actively pursued as alternatives [6,7]. Traditional tumor-related cell studies are mostly on two-dimensional (2D) culture [8-10]. However, tumors are characterized by abnormal cell growth in a complex microenvironment, and 2D cell culture is insufficient to simulate the three-dimensional (3D) growth of tumor cells *in vivo*. Tumor microenvironment simulation microfluidic chips have the advantage of controllable microenvironment and high-throughput. It can simulate tumor microenvironments and construct bionic laboratories of microfluidics [11,12].

In general, a microfluidic chip consists of micro-channels carved on substrate materials such as glass, quartz, or organic plastics silicon, which have an excellent performance. Elastic polydimethylsiloxane (PDMS), acting as the novel material for microfluidic chips, displays more advantages [13,14]. It can be made by simple methods such as soft lithography, laser etching, and injection molding which is cheap and flexible. At the same time, many researchers are still using expensive silicon or glass chips, since there is a lot of work to make reliable polymer devices [15]. The advantages and disadvantages of different chip materials are listed in Table 1. The optimal chip material will be a good choice by comprehensive consideration in the actual chip fabrication application.

**TABLE 1:**
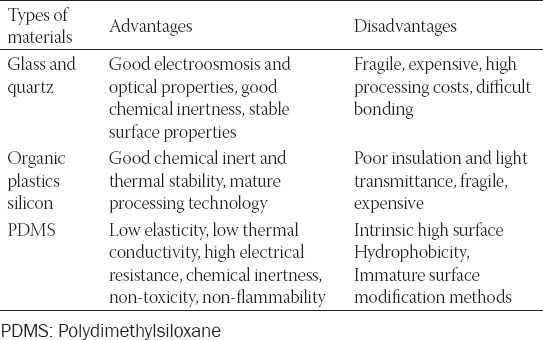
The relative merits of different chip materials

In addition to medical diagnosis [16-19], microfluidic chips are applied in broad areas including drug abuse testing [20-22], pollutant detection [22-25], counter biological weapons [26-28], and other laboratory routines. At present, microfluidic chips have been widely used in biomarkers discovery [29-31]. The application of microfluidic chip analysis technology has promoted the progress of screening for active drug compounds, which makes it possible to find effective molecules or molecular classes from a large number of natural products. The micro-analysis system is no longer at the basic application stage but is entering a relatively mature stage of industrialization and commercialization.

In this review, we summarize the recent progress of microfluidic chips in drug screening, and comprehensively compare the existing microfluidic chip screening platforms from four aspects: chip structure design, sample injection and drive system, cell culture technology on a chip, and efficient online detection technology. Furthermore, the application and developmental prospects of microfluidic chips in drug screening are discussed on the basis of chemical properties, pharmacological effects, and cytotoxicity of drugs.

## DEVELOPMENTAL HISTORY OF MICROFLUIDIC TECHNOLOGY

In a broader context, the rise of microfluidic technology is related to the development of integrated circuit technology and wafer manufacturing facilities. Richard Feynman, the winner of the Nobel Prize in Physics in 1965, envisioned the scene of manipulating and applying objects at the micro- and nano-meter levels in his famous speech “There is Plenty of Room at the Bottom” [32]. His prediction was realized by modern microelectronic technology and a variety of integrated circuit products. Simple microfluidic systems have appeared since the emergence of silicon-based micromachine technology. In 1979, Terry et al. published the first article presenting the fabrication of a complete gas chromatograph on a silicon wafer [33]. In 1990, Manz and Widmer of Ciba-Geigy Company in Switzerland were the first to improve the concept of the micro-total analysis system [34]. At that time, the concept mainly emphasized the “micro” and “full” of the analysis system as well as the Micro Electro Mechanical Systems (MEMS) processing method of the micro pipeline network, but its shape characteristics were not clear. In 1991, Manz et al. developed the capillary electrophoresis and flow injection separation on flat microchips, thus positioning the main configuration of microsystems as flat chips with a thickness of < 5 cm, with the area from several cm^2^ to more than 10 cm^2^ [35].

In 2003, the Forbes magazine listed the chip laboratory as one of the 15 most important inventions affecting the future of mankind in a special issue commemorating its 85^th^ anniversary. According to the cover article of the Business 2.0 magazine in September 2004, the chip laboratory is one of the seven technologies that will change the future. Since the concept of droplet microfluidics was proposed by the Quake Group in 2001, it has been developed and widely used in the field of high-throughput drug screening [36]. In July 2006, Nature published a series of articles on microfluidic technology, where it was introduced in detail and its important role in biomedical and pharmaceutical research was emphasized [37-41]. In July 2007, China launched the project 973 - ”Basic Research on the Application of Microfluidics in Chemistry and Biomedicine” with the continuous development of microfluidic technology. A variety of organ-on-a-chip systems, including lung chip [42-44], kidney chip [45,46], brain chip, and intestinal-liver tumor chip [47,48] were reported. These microfluidic chips can be used to simulate the interaction between different organs and tissues of the human body. Most of these organ-on-a-chip systems are constructed based on the perfusion flow model. Nowadays, the application of microfluidic technology in the field of medicine has a broad prospect, and there will be a great developmental space for the application of microfluidic technology in the research and development of new drugs.

## CONSTRUCTION OF MICROFLUIDIC CHIP PLATFORM

Drug discovery requires screening a vast number of drug candidates for their efficacy, cytotoxicity, and side effects. Screening drugs with high-throughput and high-content with low reagent consumption and cost is the focus of modern drug screening methods. At present, there are limitations in the commonly used methodologies of drug screening *in vitro*. The microfluidic chip analysis technology can miniaturize the analytic steps such as sample pretreatment, reaction, derivation, separation, and detection to a single chip; and shorten the analysis time in the form of fluid and array multi-channels. It provides a microenvironment for cell physiological and biochemical analysis with low sample consumption. The microfluidic chip analysis technology has the advantages of rapid analysis, large amounts of information, and low production cost. It provides an excellent experimental and detection technology platform for large-scale high-throughput drug screenings, especially for high-content screening of active components of drugs and natural products. In addition, microfluidic chip technology has brought new vitality to screening drug metabolites, particularly complementing virtual screening methodologies derived over the last years, thriving drug discovery as a whole [49].

The technical platform of drug screening in microfluidic chip analysis systems mainly includes chip structure design, sample injection and driving system, on-chip cell culture technology, and efficient online detection technology. Using the design technology and processing method of MEMS, micro-channel, micro-reaction chamber and other functional units can be effectively integrated into a microfluidic chip. Combining microfluidic control technology, cell culture technology, and online detection technology, microfluidic chip technology can ultimately achieve the purpose of drug screening through simulating the *in vivo* environment and analyzing the interaction between drugs and target cells.

## CHIP STRUCTURE DESIGN

In the field of microfluidic chips, there are two major types of chips based on the state of fluid supply: continuous flow chips and droplet chips. Early chip research was mostly based on the continuous flow of water. In recent years, droplet-based microfluidic chips have become the mainstream direction of microfluidic chip technology. There are two main configurations on microfluidic chips: single channel design and array channel design. Single channel design is usually used for specific functional research while the array channel design is often used for drug screening.

The simplest channel design is the cross mode [50], where the voltage is applied at both ends of the channel, and the electrophoretic separation of the sample can be carried out after micro-injection, then the online detection can be carried out by combining with laser induced fluorescence. With the development of chip processing technologies, the design of micro-channel chips has become versatile. Many chip designs with different microchannel structures and functions have emerged, such as the highly sensitive anti-parasite drug screening chips [51]. The interaction between electrically driven drugs (levamisole) and nematodes was used to detect the motor activity of nematodes for screening out the appropriate drug dose and action time. It was found that the result obtained by this chip is effective and reliable and has great development value in drug screening. Garcia et al. [52] designed microfluidic chips for screening enzyme inhibitors based on the laminar and microscale characteristics of fluorescence labeled substrates. The Y-chip can realize the rapid screening of enzyme inhibitors, which fully reflects the advantage of sample mixing brought by the microscale effect of enzyme chips. This kind of chip is frequently used in theoretical research and targeted index testing.

**FIGURE 1:**
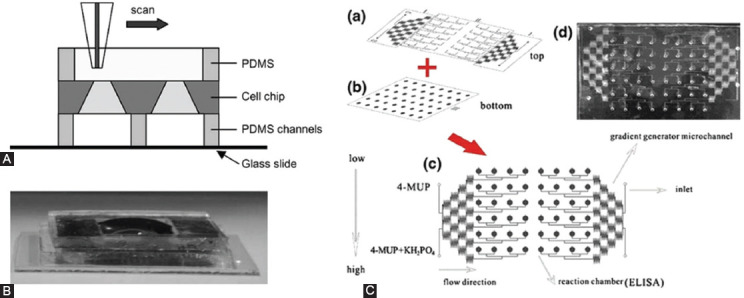
Microfluidic technologies for drug discovery. (A) Principle of scanning electrochemical microscopy measurement using a silicon chip with multi-channels of polydimethylsiloxane. The tip scanned over the collagen-cell mixture at a distance of 30 mm from the silicon substrate at a scan rate of 19 mm/s. The oxygen reduction current was monitored by the Pt microelectrode held at 0.5 V vs. Ag/AgCl; (B) Photograph of the device shown in (A). Reprinted with permission from ref [53]; (C) Schematic design of a representative gradient-generating microfluidic network. Solutions containing different chemicals were introduced from the inlets and allowed to flow through the network. The fluid streams were repeatedly combined, mixed, and split to yield distinct mixtures with distinct compositions in each of the branch channels. Reprinted with permission from ref [56].

The chip with array channel design is more commonly used in drug screenings, which is usually composed of cell chip, package cover plate, and backplane [53]. Many high-density cell arrays are arranged on the bare cell chip that are encapsulated between the cover plate and the base plate (Figure 1A and B). The cell microarray chip can accommodate all cells on the chip in the same cell cycle by controlling the cell culture conditions, so that results of biochemical and chemical reactions among different cell lines could be comparable, and multiple information can be simultaneously detected on one chip. It can better fit the purpose of high-throughput and high-content drug screening.

With the recent advances in nanoparticle magnetochemistry and redox biology, the principal focus of oxidative stress detection has been the manipulation of microchip resonator systems integrated with photolithography due to their ability to create fluid flow patterns. The electron spin resonance (ESR) on-chip technique allows for the measurement of free radicals in samples of the picoliter scale while maintaining the sensitivity of concentration measurements. Previous investigations recorded ESR spectra from 0.25 nL samples with spin concentrations of < 0.1 μM utilizing resonators with a nanoliter volume (1-3.6 nL). These devices have provided information on quantum spin diffusion at the real-space nanoscale. Furthermore, ESR signals can be detected at room temperature, which is necessary for future ESR spectroscopy on microfluidic chips combined with miniature magnets to evaluate the antitumor activity of pharmacological agents based on free radical generation in biological samples [54].

On the basis of the cell array culture chip, Won et al. [55] expanded the cell culture cavity and designed a cell array screening chip with 2100 culture cavities, in which they used to screen anticancer drugs on cell death using MCF-7 breast cancer cells. This device is more time-saving, labor-saving, and easy to use than traditional high-throughput screenings. A microfluidic chip (100 × 50 mm) designed by Kang et al. [56] for analyzing alkaline phosphatase activity using p-nitrophenyl phosphate as a substrate. The design integrates the drug concentration gradient production area, enzyme catalytic reaction area, detection area, and waste liquid pool into a chip unit (Figure 1C). The PDMS chip analysis system can screen 4 samples and 20 concentrations at a time, which fully reflects the characteristics of rapid construction of concentration gradient, high efficiency and high-throughput analysis on a microfluidic chip.

## SAMPLE INJECTION AND DRIVE

In the screening technology platform based on the microfluidic chip analysis system, the high-throughput sample injection technology mainly includes three types: array liquid storage pool, flow pool, and sampling probe sample introduction system.

Fluid driving methods can be divided into two categories. One is the use of external mechanical pumps [57,58], such as flow injection pump, pneumatic micropump, and electromagnetic micropump. This kind of method involves moving mechanical parts. It is difficult in chip processing and integration and is not commonly used. The other type is a non-mechanical driving method, which includes gravity driving [59], surface tension driving [60], and electroosmotic driving [61] that mainly rely on the external field effect to drive the flow of fluid. Huang et al. established a gravity-driven microfluidic chip flow analysis system with continuous sample change. The new sleeve-type sample change interface can realize continuous high-throughput sample introduction, and the sampling frequency can reach 80-100 samples/h [62]. By installing a drainage tube and horizontal a channel reservoir to increase and stabilize the flow rate for a long time, which not only reduces the consumption of reagents and samples, but also increases the speed and continuous working time of the system.

## ON-CHIP CELL CULTURE

Cell culture is one of the important foundations for the implementation of microfluidic chip drug screening processes. By simulating the *in vivo* environment on the chip, the morphological changes of cells are observed by fluorescence microscopy. The laser-induced fluorescence analysis technology is used to analyze drugs and cell interactions to obtain relevant information, and then to find out drug components with pharmacological activity. The microfluidic chip provides a platform for cell culture [63]. The number of cells required for the operation on the microfluidic chip is small, which is suitable for the study of cells with few but significant sources. The culture environment of the chip is similar to the one *in vivo* and can reflect the modulation between cells and extracellular matrix. This type of cell culture method is similar to the physiological state of cell microenvironment [64]. The flexible combination and integration of multiple functional units on the chip makes cell culture and analysis chip systems a great place for the process of cell sampling, culture, lysis, separation, and detection completed on the chip. On-chip cell culture can meet the analysis and monitoring requirement of high-throughput, high-content drug and cell interaction [65].

In practice, Lin et al. developed a series of 2D and 3D microgel chips with different functions, taking advantage of the micro-machining technology of permeable hydrogel materials, and applied them to localized culture of different types of cells, such as suspended leukemic cells (NB4), adherent hepatoma cells (HepG2), and fibrous cells (L929) [66]. Finally, the co-culture of different kinds of cells was realized, and good results were obtained.

## CHIP INSPECTION TECHNOLOGY

Efficient detection methods are important parts of the drug screening platforms based on the microfluidic chip analysis system. The special requirements for detection technologies include high sensitivity, fast response, miniaturization, and integration. Commonly used detectors in microfluidic chip analysis systems include fluorescence detectors [67], mass spectrometry detectors [68,69], and electrochemical detectors [70]. At present, fluorescence detection system is the most commonly used ones, which allow large-scale screening of specific molecular structures and activity under physiological conditions, track the structural changes of macromolecules with physiological functions, and achieve high-sensitivity detection on the molecular levels. Fukuda et al. [71] designed a cell culture chip with a spherical cell culture cavity to fix liver cancer cells on the culture chip and use the alkoxyresorufin O-dealkylase assay, which is a simple and useful method to evaluate drug metabolism. The appropriate drug inhibitory concentration on P450 enzymes in liver cancer cells was screened by fluorescence analysis. The advantage of this chip is that liver cancer cells are easy to culture and to be detected by fluorescence measurement.

Chen et al. [72] used microfluidic chip technology combined with Hoechst 33342/PI fluorescence technology to study the compatibility of different concentrations of alum and saltpetre on a single chip for analyzing traditional Chinese medicine components. The chip overcame the practical problems of using the traditional 96-well plate technology, such as the tedious operation of single-dye methods. Moreover, the treated chip can be reused, and thus saves reagents and experimental consumables. The dynamic culture of cells under the microscale conditions is closer to the real physiological conditions of cells, which makes the experimental results reliable.

The screening technology of microfluidic chips shows great advantages over the previous screening technologies, but it is still in the stage of development, and there are still a large number of technical problems to be solved.

## SCREENING NATURAL PRODUCT ANTICANCER DRUGS

Natural products have made significant contributions to cancer chemotherapy in the past a few decades and are still an indispensable source of anticancer drug discovery today. Natural products can be regarded as a class of dominant structures formed through long-time evolution in nature that can preferentially interact with a variety of drug target proteins to produce specific activities. Compared to synthetic compounds or combinatorial chemistry libraries, natural products are diverse in both structural framework and stereochemistry. They usually have a broad range of molecular weights and lipo-water distribution coefficients that are very similar to those of marketed drugs. With the development of economy and the modification of raw materials, the negative impact of chemicals on human health and the environment has been highly valued, and the trend of returning to nature has emerged. Therefore, the demand for natural medicines in the international market is increasing. In addition, as combinatorial chemistry and high-throughput screening productivity reduced, the strict examination and approval of new drugs, and the rising costs of drug research and development, people began to realize the importance of natural products. The novel skeletons and excellent biological activity of natural products occupies critical shares in drug research and development. The use of natural products as sources of drug research and development is on the rise [73,74].

**FIGURE 2:**
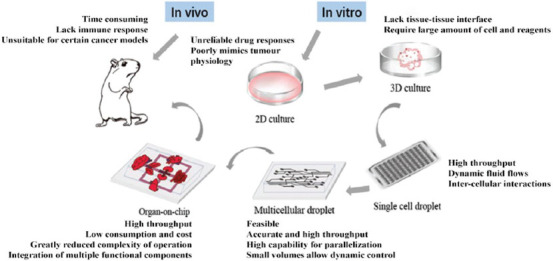
Microfluidic technologies for drug discovery. Schematic diagram showing common chip types and preclinical models for anticancer drug screening research. Compared with the conventional drug screening methods based on Petri dishes and experimental animals, microfluidic cell culture have many advantages including miniaturized size, good repeatability, ease-to-use, high sensitivity, and high-throughput, and it can be used for screening a great number of drugs at different concentrations.

With the improvement of drug isolation, detection, and high-throughput drug screening methodologies, international pharmaceutical researchers will face more challenges, including improving the efficiency of drug discovery from natural products, reducing the cost of discovery, and obtaining the desired active ingredients [75]. Combining high-throughput screening with microfluidic chip technologies, the application of active natural product screening can not only simultaneously evaluate the activity and toxicity of natural products but also greatly reduce the number of animal experiments, thus speeding up the research and development of natural anticancer drugs (Figure 2). The applications of microfluidic chips in drug screening were summarized in Table 2.

**TABLE 2:**
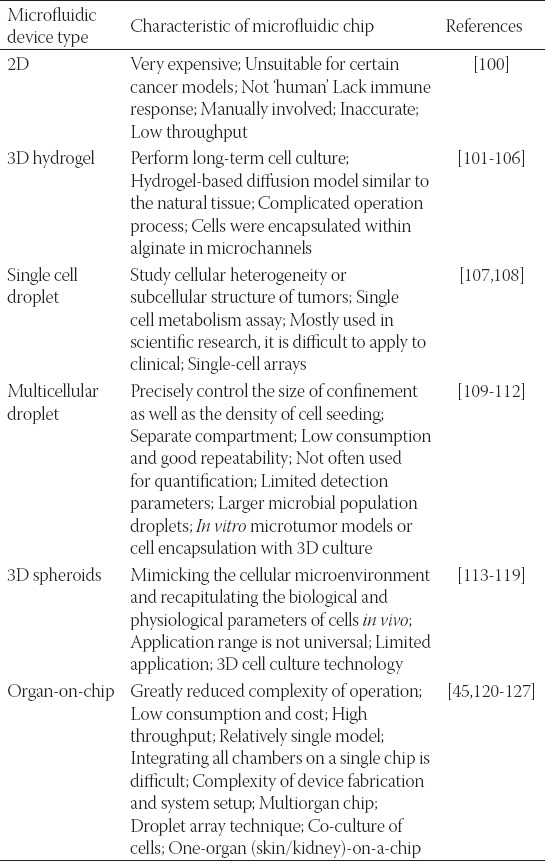
Applications of microfluidic chips in drug screening

## SCREENING BASED ON THE CHEMICAL PROPERTIES OF DRUGS

Most extracts of traditional Chinese medicine are mixtures of various complex components, and the active components with different structures have different characteristics. Modern analytical chemistry techniques represented by liquid chromatography-spectroscopy are applied to the separation, extraction, and analysis of natural products that provide essential information for the screening of active components.

Lin et al. [76] incorporated a porous polymer monolithic microcolumn into a simple microfluidic chip, which was used for the separation and determination of catechins in green tea by solid phase microextraction and chemiluminescence detection. The microfluidic chip can realize online enrichment detection without gradient elution and has the advantages of reusability, high sensitivity, and low reagent consumption. Combined fluid injection with capillary electrophoresis and based on the principle of microfluidic chip, Cheng et al. [72] designed an H-type microfluidic chips and successfully used it for the separation and detection of four alkaloids: sophorine, sophocarpine, matrine, and oxymatrine. The H-type microfluidic chip has the advantages of good separation, speed, and accuracy. It provides an effective and reliable method for rapid separation and detection of active components of natural products. Zhao et al. [77] designed a large-capacity high-performance liquid phase chip coupling device and used tiny analysis columns to separate substances of different polarity. The UV detection sensitivity of this chip was 4-30 times higher than that of LC-MS, which provided an effective method for the separation and detection of natural components and complex compounds of degradation products in industry.

## SCREENING BASED ON THE PHARMACOLOGICAL ACTION OF DRUGS

One of the foundations of high-throughput drug screening is the interaction between drugs and biomacromolecules. Based on the combination of microfluidic chip technology and high-throughput screening, Wu et al. [78] created an array high-throughput screening chip with “sandwich” structure at the cellular level. The potential anticancer drugs were screened by fluorescence detection based on the interaction between drugs and breast cancer cells (MCF-7). This method provides a fast and low-cost way for screening the active components of natural products. On the basis of the traditional chip, Zheng and others added a microvalve to control the microfluidic fluid flow, and used SU8 negative photoresist mold and PDMS to create a double-layer microfluidic cell array chip [79]. The chip intercepts sample cells in a fixed area of the cell culture chip through the C-type dam structure, and binds to the double-layer PDMS to form a valve control layer. The switching function of the valve network successfully enables the control of the fluid flow in the chip channel. Simultaneously, the chip provides a drug concentration gradient network to produce six different concentrations of drugs for cell stimulation. The feasibility of the chip in cell research and drug screening was verified by the detection of the drug activity on three kinds of co-cultured cells and the gradient concentration of irinotecan (CTP11) on hepatocellular carcinoma cells.

Liu et al. designed a microfluidic chip with parallel cell culture chamber, which contains four parallel cell culture chambers and independent sample entrance, outlet, and channel [80]. The rat C6 glioma cells were successfully cultured in the cell culture chamber for 7 days. On this basis, the cells were labeled with fluorescence and treated with different concentrations of colchicine. The effects of different concentrations of colchicine on the cells were obtained by observing the morphological changes of the cells by drug exposure. The results showed that the cell culture chip can provide reliable methods for cancer research. In addition, Popovtzer et al. designed an electrochemical detection chip to detect the effect of different concentrations of hydroxyisobutyric acid on the activity of alkaline phosphatase in colon cancer cells. The results showed that this detection device is fast, sensitive, miniaturized, and suitable for high-throughput analysis and screening [81].

## SCREENING BASED ON CYTOTOXICITY OF DRUGS

The metabolic pathways and cytotoxicity of drugs in cells are also important aspects of high-throughput screenings. Microfluidics can provide advanced *in vitro* systems that combine liver tissue or cells to study the metabolism and toxicity of drugs [82-84]. Ma et al. developed a microfluidic device and verified the feasibility of drug metabolism study on the microfluidic chip through experiments on acetaminophen (AP) UDP-glucuronyltransferase (UGT) metabolism and its cytotoxic effect on HepG2 cells [85].

Mao et al. have developed a microfluidic device, combined with mass spectrometry, that mimics drug metabolism in the human liver and its cytotoxicity to cells [86]. Sugiura et al. designed an array cell culture chip by the use of independent perfusion channels and a pressure-driven fluid transmission system. In this chip, each cell culture cavity has an independent perfusion channel, thus avoiding the interaction between adjacent channels, and to carry out parallel experiments and drug screenings [87]. HeLa cells were labeled with fluorescence and the status of cells was observed microscopically. The cytotoxicity of carboplatin, cisplatin, doxorubicin, etoposide, paclitaxel, and tamoxifen was tested. The cell response to the drugs was also successfully observed. The chip can easily remove the air foam, uniform injection, and quantify cell proliferation, which provides an effective method for the screening of drug candidates. The integration and flowing liquid pressure injection of the device can enable it to achieve large-scale high-throughput drug screening.

**FIGURE 3:**
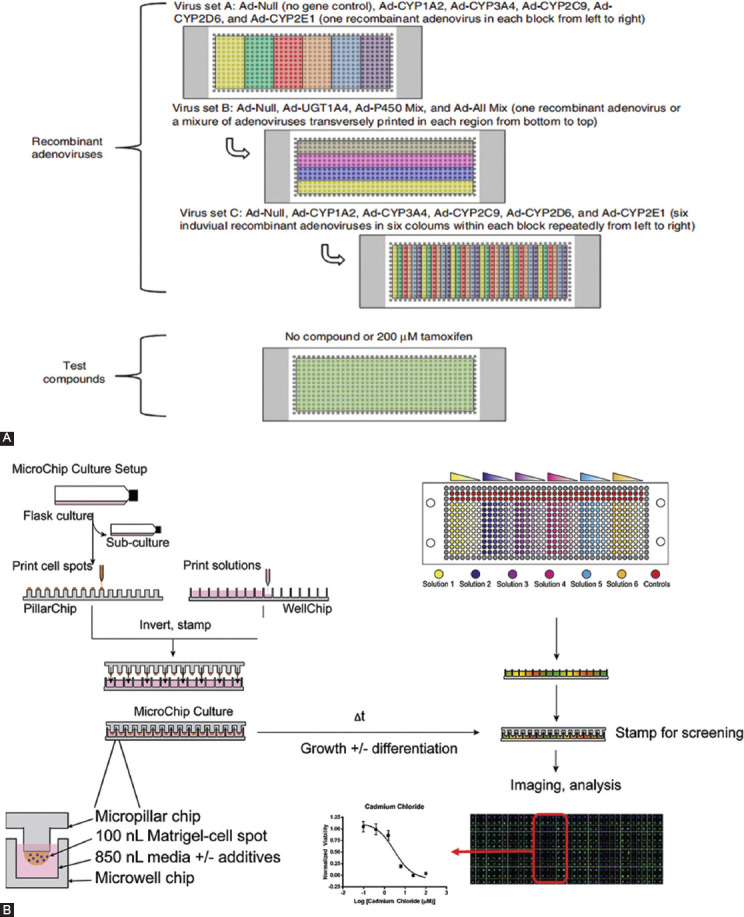
Microfluidic chip based on cytotoxic screening. (A) Layout of the microwell chip containing 84 combinations of multiple recombinant adenoviruses (three sets of recombinant adenoviruses dispensed sequentially) to prepare the TeamChip for high-throughput gene transduction, and an additional microwell chip containing 200 mM tamoxifen for metabolism-induced toxicity screening. Reprinted with permission from ref [89]; (B) The platform consists of two complementary polystyrene microchips. Cell spots consist of Matrigel-encapsulated cells spotted as 100 nL cultures atop micropillars. Medium change consists of lifting a PillarChip from one WellChip and stamping into another containing fresh medium. Fluorescence-based endpoint assays are used to measure viability, proliferation, and protein expression of on-chip cultures. Reprinted with permission from ref [90].

Wang et al. designed a multi-level rubber array (24 × 24) microfluidic high-throughput screening chip [67]. Mammalian cells were labeled with fluorescence and observed under a fluorescence microscope. Through the interaction between drugs and cells, digitalis saponins, saponins, acrolein, and other toxic substances were screened by three different types of cells (BALB/3T3 cells, HeLa cells, and bovine endothelial cells). The effects of toxic substances on the morphology and viability of cells were observed, and a high-density parallel drug screening method was provided. For spatial screening of multiple compounds, Lee et al. developed a small 3D cell culture array that consists of human cells encapsulated with collagen or alginate gels. It can be used for high-throughput toxicity screening of drug candidates and their cytochrome P450-generated metabolites [88]. Specifically, the microchips can be cultured to support cell growth on time scales associated with toxicity analysis and be used to detect drug metabolites and evaluate the cytotoxicity of metabolites at the same time. The above studies suggested that microfluidic chip can be used to study drug metabolism and other complex processes. Kwon et al. developed the “Transfected Enzyme and Metabolism Chip (TeamChip),” which is composed of two complementary chips. This particular chip could carry out 3D cell culture microfluidics on the nanoscale for rapid screening on toxicity of compounds using human hepatocellular cancer cells (Figure 3A) [89]. Nierode et al. used this microfluidic platform for high-throughput screening. The human neural progenitor cell lines maintain their pluripotent state and can be induced for differentiation. The resulting cultures are used to quantify the expression of key cell proteins, to screen for acute toxicity and anti-diffusion effects of various bioactive compounds, and to investigate the existence of variable toxicity sensitivity between undifferentiated and differentiated cells (Figure 3B) [90].

## SCREENING DRUGS BASED ON TISSUE HYPOXIA

Heterogeneity of hypoxia in tumors and their microenvironments is a critical barrier to radiotherapy and chemotherapy as it drives tumor progression and leads to therapeutic resistance [91]. In addition to fine temporal and spatial control of oxygen, on-chip cultures can be coupled with real-time monitoring such as optical microscopy, fluorescence, nuclear magnetic resonance, and ESR [92,93]. Such microfluidic devices revealed that tumor spheroids changed their size and shape under hypoxic conditions. A combination of precise oxygen control, drug delivery, and two-photon imaging in a single microfluidic chip showed significant difference in doxorubicin uptake between 0% and 20% oxygen profiles. Further analysis of data collected from the device showed heterogeneous uptake of the chemotherapeutic agent among individual tumor cells [94-96]. Microfluidics can also be set for the assessment of magnetic nanotheranostic approaches targeting tumor hypoxia [97], in which magnetic fields guide magnetic nanoparticles and modulate the kinetics of redox reactions between the nanoparticles and the antitumor drugs in tumor models [98,99].

## CONCLUSION

Microfluidic chip analysis is one of the frontier fields of science and technology. As a new technology platform for cell research, it has the advantages of high integration, high sensitivity, high throughput, less reagent consumption, and prompt large-scale analysis. Microfluidic chip can realize the miniaturization, automation and portability of whole analysis and test equipment. On the other hand, the micron-scale channel structure in the chip can accurately control substance concentration gradient and microfluidic liquid flow, adjust cell microenvironment elements such as solution temperature and pH, simulate cell growth microenvironment, and complete real-time monitoring. Microfluidic chips have been widely used in the study of cell behaviors. Its models are divided into 2D and 3D categories. Combining the perspective of plane culture with three-dimensional growth, the effects of different drug concentration gradients, electrical stimulation, or cell-cell interaction on cell behaviors are studied, breaking the limitations of traditional methods.

Microfluidic analysis systems have developed from capillary electrophoresis separation, such as liquid-liquid extraction, filtration, and membrane-free diffusion. The multiphase laminar flow separation of microfluidic has the advantages of simple structure, diversified separation function, and great application potential. Compared to the traditional analytical methods, the microfluidic chip applied to the separation and detection of natural products can make the pretreatment process of natural products easier, faster, and cheaper. It is also easy to combine with other devices, such as spectroscopy, chromatography and mass spectrometry, which can be used to study drug metabolism in cells.

To date, although the role of microfluidic chips in drug discovery has been recognized and refined, microfluidic technology has not been able to replace traditional drug screening platforms. At present, the research of microfluidic chips has been pretty in-depth. However, due to the variety of microfluidic chip designs and the lack of relevant industrial support, the cost of chips is high and it is difficult to promote to various laboratories. Therefore, even though microfluidic chips have broad application prospects and highly potential application value, there are great challenges in the industrialization and popularization of microfluidic chips.
